# Laryngotracheal Microbiota in Adult Laryngotracheal Stenosis

**DOI:** 10.1128/mSphereDirect.00211-19

**Published:** 2019-05-01

**Authors:** Alexander T. Hillel, Sharon S. Tang, Camila Carlos, Joseph H. Skarlupka, Madhu Gowda, Linda X. Yin, Kevin Motz, Cameron R. Currie, Garret Suen, Susan L. Thibeault

**Affiliations:** a Department of Otolaryngology–Head and Neck Surgery, Johns Hopkins University School of Medicine, Baltimore, Maryland, USA; b Department of Surgery, University of Wisconsin–Madison, Madison, Wisconsin, USA; c Department of Bacteriology, University of Wisconsin–Madison, Madison, Wisconsin, USA; d Department of Otorhinolaryngology, Mayo Clinic, Rochester, Minnesota, USA; DOE Joint Genome Institute; University of California, San Francisco; Cincinnati Children's Hospital Medical Center

**Keywords:** *Acinetobacter*, *Moraxella*, fibrosis, laryngotracheal stenosis, subglottic stenosis, upper airway microbiota

## Abstract

Laryngotracheal stenosis is an obstructive respiratory disease that leads to voicing difficulties and dyspnea with potential life-threatening consequences. The majority of incidences are due to iatrogenic etiology from endotracheal tube intubation; however, airway scarring also has idiopathic causes. While recent evidence suggests a microbial contribution to mucosal inflammation, the microbiota associated with different types of stenosis has not been characterized. High-throughput sequencing of the V4 region of the16S rRNA gene was performed to characterize the microbial communities of 61 swab samples from 17 iatrogenic and 10 adult idiopathic stenosis patients. Nonscar swabs from stenosis patients were internal controls, and eight swabs from four patients without stenosis represented external controls. Significant differences in diversity were observed between scar and nonscar samples and among sample sites, with decreased diversity detected in scar samples and the glottis region. Permutational analysis of variance (PERMANOVA) results revealed significant differences in community composition for scar versus nonscar samples, etiology type, sample site, groups (iatrogenic, idiopathic, and internal and external controls), and individual patients. Pairwise Spearman’s correlation revealed a strong inverse correlation between *Prevotella* and *Streptococcus* among all samples. Finally, bacteria in the family *Moraxellaceae* were found to be distinctly associated with idiopathic stenosis samples in comparison with external controls. Our findings suggest that specific microbiota and community shifts are present with laryngotracheal stenosis in adults, with members of the family *Moraxellaceae*, including the known pathogens *Moraxella* and Acinetobacter, identified in idiopathic scar. Further work is warranted to elucidate the contributing role of bacteria on the pathogenesis of laryngotracheal stenosis.

**IMPORTANCE** The laryngotracheal region resides at the intersection between the heavily studied nasal cavity and lungs; however, examination of the microbiome in chronic inflammatory conditions of the subglottis and trachea remains scarce. To date, studies have focused on the microbiota of the vocal folds, or the glottis, for laryngeal carcinoma, as well as healthy larynges, benign vocal fold lesions, and larynges exposed to smoking and refluxate. In this study, we seek to examine the structure and composition of the microbial community in adult laryngotracheal stenosis of various etiologies. Due to the heterogeneity among the underlying pathogenesis mechanisms and clinical outcomes seen in laryngotracheal stenosis disease, we hypothesized that different microbial profiles will be detected among various stenosis etiology types. Understanding differences in the microbiota for subglottic stenosis subtypes may shed light upon etiology-specific biomarker identification and offer novel insights into management approaches for this debilitating disease.

## Abstract (precis)

The laryngotracheal region resides at the intersection between the heavily studied nasal cavity and lungs; however, examination of the microbiome in chronic inflammatory conditions of the subglottis and trachea remains scarce. To date, studies have focused on the microbiota of the vocal folds, or the glottis, for laryngeal carcinoma, as well as healthy larynges, benign vocal fold lesions, and larynges exposed to smoking and refluxate. In this study, we seek to examine the structure and composition of the microbial community in adult laryngotracheal stenosis of various etiologies. Due to the heterogeneity among the underlying pathogenesis mechanisms and clinical outcomes seen in laryngotracheal stenosis disease, we hypothesized that different microbial profiles will be detected among various stenosis etiology types. Understanding differences in the microbiota for subglottic stenosis subtypes may shed light upon etiology-specific biomarker identification and offer novel insights into management approaches for this debilitating disease.

## INTRODUCTION

Laryngotracheal stenosis induces airway constriction via scar tissue formation, which negatively affects voicing and communicative abilities, compromises respiratory support, and may lead to critical, life-threatening complications (1–4). Depending on the severity of the presenting stricture, symptoms can range from mild dyspnea upon physical exertion, dysphonia, and stridor up to major respiratory distress due to airway obstruction (1, 3, 4). The principal anatomic location is the subglottis and/or proximal trachea; however, scarring may involve the posterior glottis at the level of the vocal folds or, less frequently, the supraglottis (1, 5, 6, 49). As such, laryngotracheal stenosis is also termed subglottic stenosis. The majority of subglottic stenosis incidences are associated with iatrogenic causes, such as intubation injury or tracheotomy, that provoke epithelial damage; however, scar bands may also form as a result of autoimmune disease or focal trauma or remain idiopathic in etiology for scars occurring with unknown cause (1, 2, 5, 7, 8). While these stenosis subtypes encompass differing etiologies for pathogenesis, population types, and clinical outcome consequences, mucosal inflammation is a shared characteristic among all stenosis types (1).

At the crux of stenosis lies an aberrant healing mechanism. Injuries sustained in the epithelium can lead to subepithelial inflammation (1, 9). In the tracheal mucosal lamina propria, inflammation triggers the proliferation of fibroblasts that produce extracellular matrix proteins that contribute to subsequent granulation tissue deposition and scar formation (1, 9). In the case of laryngotracheal stenosis, this subepithelial inflammation is suspected to be T lymphocyte mediated, with an imbalance toward profibrotic cytokines contributing to the excessive deposition of scar tissue (2, 10, 11). Histologic studies specific to laryngotracheal stenosis have revealed increased vascularization within granulation tissue, fibrosis with thickened collagen bundling, mucus duct and gland atrophy, and inflammatory ulceration (1, 9). Because many inflammatory diseases in other bodily regions have known bacterial connections, it has long been speculated that a microbial contribution exists for this stenosis-affiliated inflammation, with early work demonstrating links between general bacteriologic pathogenesis via respiratory infections and iatrogenic stenosis (12–14). Further advances in molecular technologies have allowed for investigations of mucosal inflammatory factors, revealing an aberrant upregulation in inflammatory pathway interleukin-17A (IL-17A)/IL-23 within idiopathic subglottic stenosis mucosa (10). Since the primary producer of IL-17A is a subset of T cells (γδ T cells) known to recognize microbial antigens, the role of microbes in stenotic disease development is highly implicated (10).

As recent advancements in next-generation sequencing allow for whole microbial community profiling, the understanding of the role of the microbiome in human health and disease states has expanded our understanding of the microbial ecology of the upper and lower respiratory tracts (15, 16). Many studies have focused on chronic inflammatory conditions in the lungs and nasal cavity, demonstrating changes in community diversity and associations between specific microbial strains and disease (17–20). Although the laryngotracheal region resides at the intersection between these heavily studied sites, examination of the microbiome of this area remains scarce. To date, studies have focused on the microbiota of the vocal folds, or the glottis, for laryngeal carcinoma as well as healthy larynges, benign vocal fold lesions, and larynges exposed to smoking and refluxate (21, 22).

The trachea is anatomically adjacent and connected to the inferior aspect of the glottis. These tissues are composed of two different types of epithelial cells, with stratified squamous epithelium and ciliated pseudostratified columnar epithelium lining the vocal folds and the trachea, respectively (15). Recent work specific to laryngotracheal stenosis has shown *Mycobacterium* species to be uniquely associated with idiopathic subglottic scar patients and Acinetobacter species to be PCR positive in iatrogenic subglottic stenosis (23). Using a PCR array to analyze 10 idiopathic stenosis samples and 10 iatrogenic stenosis samples, this study offered initial evidence suggesting potential differences in microbial representation among these proximal sites in the airway.

Here we sought to further examine the entire microbial community composition and structure, using 16S rRNA amplicon sequencing, of adult laryngotracheal scars of various etiologies. Due to the heterogeneity among the underlying pathogenesis mechanisms and clinical outcomes seen in laryngotracheal stenosis disease, we hypothesized that different microbial profiles will be detected among various stenosis etiology types. Based on previous work of the pathological vocal fold and laryngotracheal tissues, we anticipated identifying similar etiology-dependent pathogen associations; we also expected decreases in species diversity in stenosis samples in comparison with that of nonstenosis regions both within patients with stenosis and in patients without stenosis. Understanding differences in the microbiota for subglottic stenosis subtypes may shed light upon etiology-specific biomarker identification and offer novel insights into management approaches for this debilitating disease.

## RESULTS

### Disease etiologies.

A total of 39 patients were recruited, with 21 of those presenting with iatrogenic scarring, 10 with idiopathic scarring, and 8 control patients with benign vocal fold lesions but no subglottic or tracheal pathology. Of the 121 swab samples collected, 61 samples yielded adequate sequences after bioinformatics processing for subsequent statistical analysis. Of these 61 swab samples, 19 were collected from iatrogenic scars, 8 were from idiopathic scars, 27 were from healthy parts of the trachea in patients with stenosis (internal controls), and 7 were from normal tracheas in external control patients. These samples represented 27 patients with laryngotracheal stenosis (17 from iatrogenic scars, 10 from idiopathic scars) and 4 patients with vocal fold disease serving as laryngotracheal stenosis-free external controls. Disease and demographic characteristics are provided in Table S1 in the supplemental material.

10.1128/mSphereDirect.00211-19.3 TABLE S1 Detailed metadata for all swab samples. Internal control samples are denoted as Int control, while external controls are marked as Ext control. Download Table S1, PDF file, 0.1 MB. Copyright © 2019 Hillel et al. 2019 Hillel et al. This content is distributed under the terms of the Creative Commons Attribution 4.0 International license.

### Sequencing results.

**FIG 1:**
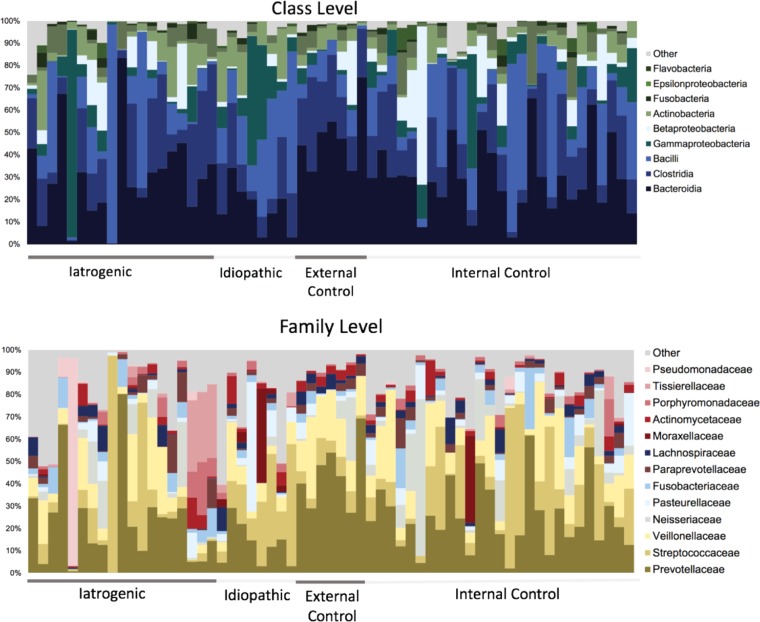
Taxonomic composition across all disease groups. Relative abundance for class and family level representation among all samples inclusive of all disease etiologies.

After bioinformatics processing in mothur, a total of 2,795,075 raw sequences averaging 25,383 sequences per sample yielded 2,231,962 high-quality sequences. Samples had a mean of 37,968 sequences (range, 1,922 to 127,612). Only samples with >97% coverage were included in subsequent analysis, with resulting Good’s coverage averaging 99% and an apparent leveling of rarefaction curves (Fig. S1), indicating sufficient sampling for all included samples. After subsampling, a total of 2,388 operational taxonomic units (OTUs) at 97% similarity were identified across all samples and classified into the following phyla, in order of abundance: *Bacteroidetes* (35%)*, Firmicutes* (33%)*, Proteobacteria* (9.5%)*, Actinobacteria* (6.5%), and *Spirochaetes* (3%). Other phyla identified at low abundances (<2%) include the *Tenericutes* (1.26%), *Verrucomicrobia* (0.9%), candidate phylum SR1 (0.45%), and *Synergistetes* (0.36%). Nine classes of bacteria were represented among the samples, with the top five consisting of the *Bacteroidia*, *Clostridia*, *Bacilli*, *Gammaproteobacteria*, and *Betaproteobacteria*. At the family level, the samples were dominated by the following top five families: *Prevotellaceae*, *Streptococcaceae*, *Veillonellaceae*, *Neisseriaceae*, and *Pasteurellaceae* (Fig. 1).

10.1128/mSphereDirect.00211-19.1 FIG S1 Rarefaction curves for all samples. Rarefaction curves demonstrating sequencing plateau for all samples, indicating adequate sampling coverage. Download FIG S1, EPS file, 2.5 MB. Copyright © 2019 Hillel et al. 2019 Hillel et al. This content is distributed under the terms of the Creative Commons Attribution 4.0 International license.

### Correlations among genera.

**FIG 2:**
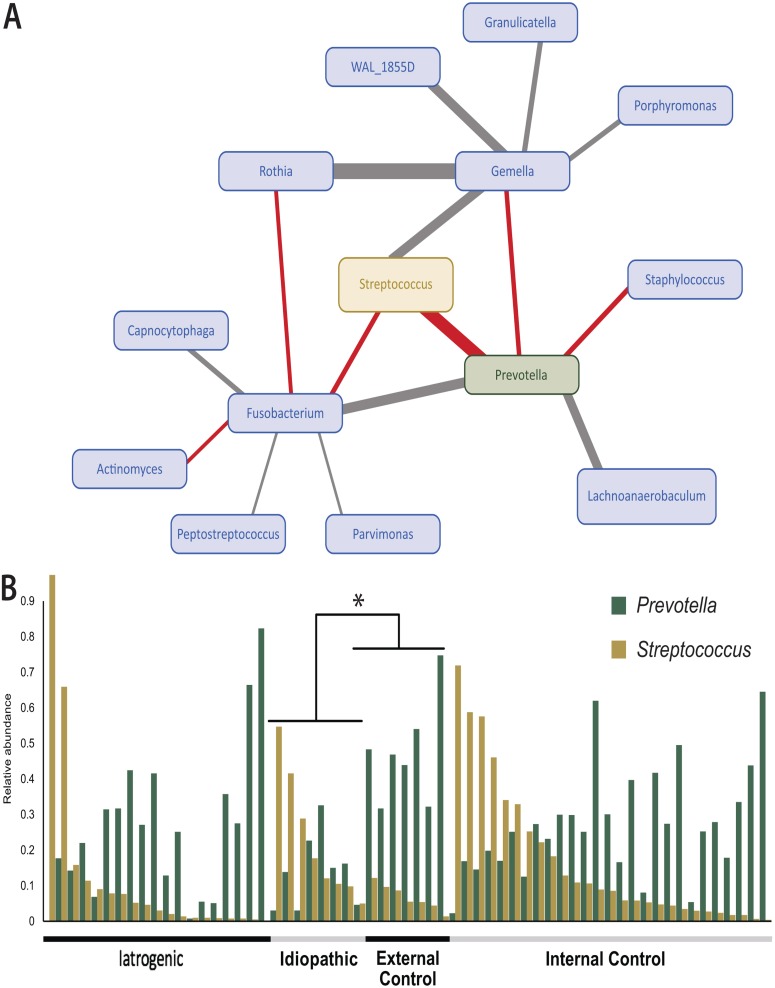
Correlations in genera identified in all samples. (A) Network of nodes depicting directional relationships among the 14 bacterial genera identified to be statistically significantly correlated in the top 15 genera, with negative correlations depicted with red lines and positive correlations depicted with gray lines. The strength of correlation determined via the Spearman rank correlation test is depicted by the thickness of the connecting lines between genus nodes. (B) The negative correlation between *Prevotella* and *Streptococcus* is further illustrated in relative abundance patterns among all samples sorted by group, with *Prevotella* depicted in green and *Streptococcus* depicted in yellow.

Pairwise Spearman’s correlation was used to determine correlations among the top 15 identified genera. Significant correlations (*P < 0.05*) were identified for 14 genera, with a notably strong inverse correlation between *Prevotella* and *Streptococcus* (Fig. 2 and Table 1).

**TABLE 1:** Spearman’s rank correlations for significant genus pairs

Genus pair correlation	Rho value
*Streptococcus-Prevotella*	−0.42988
*Streptococcus-Fusobacterium*	−0.28635
*Streptococcus-Gemella*	0.34186
*Prevotella-Staphylococcus*	−0.28576
*Prevotella-Gemella*	−0.28715
*Prevotella-Lachnoanaerobaculum*	0.32588
*Prevotella-Fusobacterium*	0.38682
*Gemella-Porphyromonas*	0.29951
*Gemella-Granulicatella*	0.30396
*Gemella-WAL_1855D*	0.3962
*Gemella-Rothia*	0.4569
*Fusobacterium-Actinomyces*	−0.27893
*Fusobacterium-Parvimonas*	0.26513
*Fusobacterium-Peptostreptococcus*	0.26801
*Fusobacterium-Capnocytophaga*	0.30239

### Comparison of microbial communities in stenosis etiologies and control groups.

Community diversity and richness were measured using the Shannon, Chao, dominance, and evenness indices (Table 2) and assessed for differences using Kruskal-Wallis testing with Bonferroni’s multiple comparison correction. Statistically significant differences in the distribution of taxa were detected between scar and nonscar tissue types among all etiology groups, along with differences in richness, as measured by Chao’s index (*P = *0.021). Scar tissue samples demonstrated decreased diversity compared with nonscar tissue samples, with average Chao’s values of 66.83 ± 82.86 and 118.28 ± 146.05, respectively. Further, differences were found in community evenness and richness for sample sites inclusive of all etiology groups (*P* = 0.009), with *P* values of 0.026 and 0.010 for evenness and Chao’s indices, respectively. Mean evenness and Chao’s values indicate less diversity at the glottic region (Table 3). There was greater abundance of *Prevotella* in external mucosal controls than in idiopathic stenosis specimens (*P* = 0.003). No significant differences in diversity were noted among samples categorized by stenosis etiology (iatrogenic stenosis, idiopathic stenosis, and nonstenosis), and this remained consistent when sample site was factored in with etiology to assess differences in groups (iatrogenic stenosis, idiopathic stenosis, internal control, and external control) as reflected by principal-component analysis (PCA) of all individual samples (Fig. S1).

**TABLE 2:** Community diversity by group as measured by Shannon, Chao, dominance, and evenness metrics^*a*^

Group	Shannon index	Chao index	Dominance index	Evenness index
Iatrogenic stenosis	2.46 ± 1.11	72.2 ± 98.6	0.23 ± 0.27	0.32 ± 0.13
Idiopathic stenosis	2.6 ± 0.65	51.08 ± 15.29	0.18 ± 0.12	0.35 ± 0.16
Internal control	2.7 ± 0.85	123.76 ± 159.35	0.18 ± 0.13	0.24 ± 0.07
External control	2.50 ± 0.35	97.15 ± 81.41	0.17 ± 0.06	0.27 ± 0.10

a Mean value ± standard deviation was obtained for each index relative to group. No statistical differences were noted by group, where internal controls represented disease-free regions from patients with stenosis and external controls represented disease-free regions from patients without stenosis.

**TABLE 3:** Community diversity by sample site as measured by Shannon, Chao, dominance, and evenness metrics^*a*^

Sample site	Shannon index	Chao index	Dominance index	Evenness index
Glottis	1.28 ± 1.11	31.9 ± 19.81	0.53 ± 0.37	0.19 ± 0.10
Subglottis	2.75 ± 0.76	75.42 ± 94.35	0.15 ± 0.11	0.35 ± 0.13
Supraglottis	2.77 ± 0.83	140.85 ± 168.44	0.17 ± 0.12	0.26 ± 0.09
Trachea	2.51 ± 0.58	64.8 ± 30.29	0.18 ± 0.12	0.30 ± 0.11

a Mean value ± standard deviation was obtained for each index relative to sample site. Reduced diversity in community members is noted for the glottis region compared to that of other regions sampled.

Differences in community composition were evaluated using a permutational analysis of variance (PERMANOVA) test for Bray-Curtis dissimilarity values based on the following variables: patient, sex, smoking status, tissue type, etiology, sample site, and group. Significant differences found among these factors are shown in Table 4 with *post hoc* analyses completed (Table S2). Fisher’s exact test (*P = *0.021) revealed that the *Moraxellaceae* family, which includes the genera *Moraxella* and Acinetobacter, is more frequently found in the idiopathic samples (5 of 7 samples) than in the external controls (0 of 7 samples).

**TABLE 4:** PERMANOVA for etiology groups and variables

PERMANOVA variable	*P* value^*a*^
Group (iatrogenic, idiopathic, mucosal control, stenosis control)	0.0008
Tissue type (scar, nonscar)	0.0346
Etiology (iatrogenic stenosis, idiopathic stenosis, nonstenosis)	0.0113
Sample site (supraglottis, glottis, subglottis, trachea)	0.0077
Sex (female, male)	0.0246
Smoking status (smoking history, nonsmoker)	0.0545
Patient (comparisons among individual patients)	0.0001

a *P* values of <0.05 indicate statistical significance.

10.1128/mSphereDirect.00211-19.4 TABLE S2 Results for permutational analysis of variance of Bray-Curtis dissimilarity for all samples with post hoc analyses. PERMANOVA results and post hoc analyses for the following variables: (A) group (iatrogenic stenosis, idiopathic stenosis, mucosal control, stenosis control); (B) tissue type (scar tissue, nonscar tissue); (C) etiology (iatrogenic stenosis, idiopathic stenosis, nonstenosis/vocal fold disease); (D) sample site (supraglottis, glottis, subglottis, trachea); (E) sex (male, female); (F) smoking status (smoker/smoking history, nonsmoker); (G) patient (interpatient comparison). Download Table S2, PDF file, 0.2 MB. Copyright © 2019 Hillel et al. 2019 Hillel et al. This content is distributed under the terms of the Creative Commons Attribution 4.0 International license.

### OTU differences between stenosis samples and external controls.

**FIG 3:**
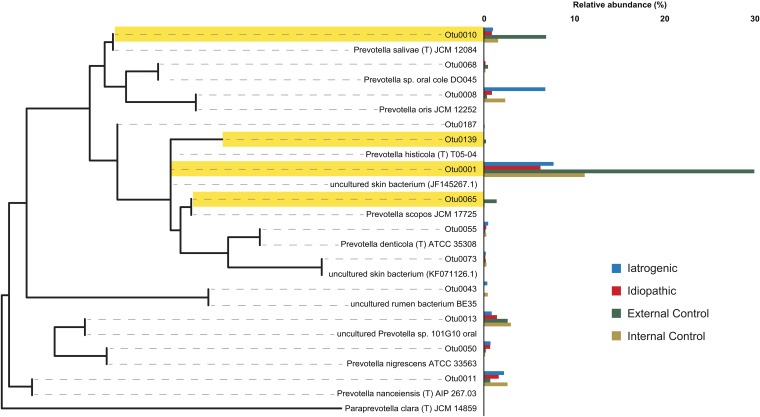
*Prevotella* phylogenetic tree featuring OTUs distinctly abundant in mucosal control samples versus stenosis samples. OTUs highlighted in yellow illustrate the OTUs identified as significantly different between mucosal controls versus stenosis groups and are coupled with relative abundance percentages for all *Prevotella* OTUs identified among iatrogenic stenosis (blue), idiopathic stenosis (red), external (green), and internal control (yellow) groups. Although OTU 0187 was identified as significantly represented in external controls, it was excluded from highlighting due to very low relative abundance levels.

**FIG 4:**
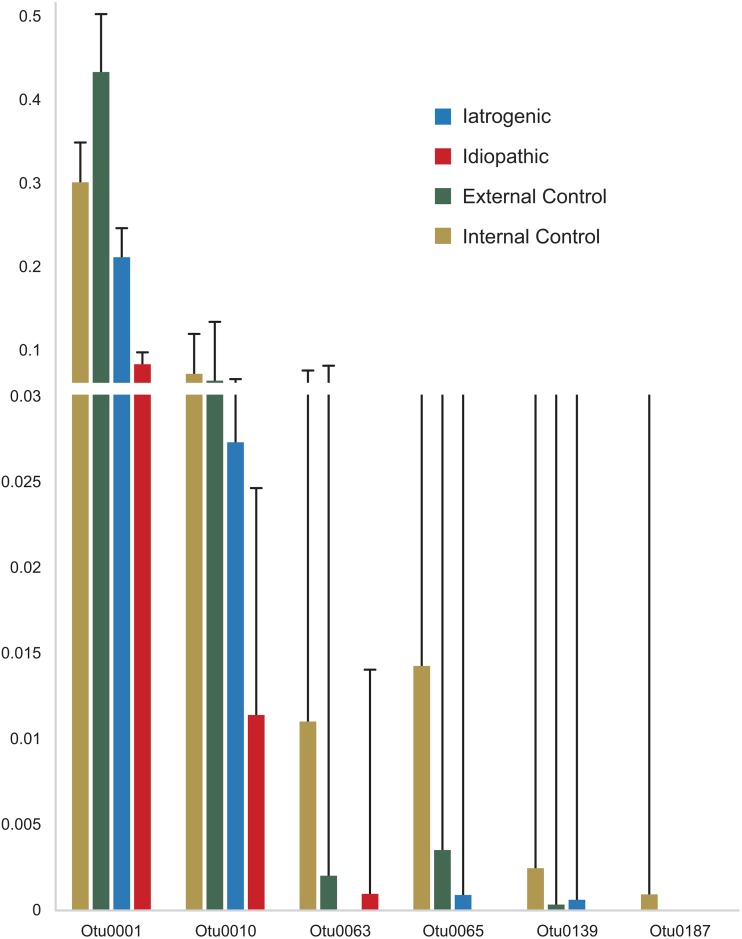
Proportion of significant OTUs by group type. Proportions of the six significantly identified OTUs among etiology groups. External controls (illustrated in yellow) demonstrate significantly higher proportions of all six OTUs than other group types.

Six OTUs (0001, 0010, 0063, 0065, 0139, 0187) representing *Leptotrichia* (0063) and *Prevotella* (all other OTUs) were identified as having statistically different abundances among groups (iatrogenic stenosis, idiopathic stenosis, internal control, external control) (Fig. 3). These genera are members of the normal oral and gut communities (24–27) and are markedly lacking within the iatrogenic stenosis group and the idiopathic stenosis group, although they are well represented in external and internal controls (Fig. 4).

## DISCUSSION

Here we utilize next-generation sequencing to characterize the microbiota of patients with iatrogenic and idiopathic laryngotracheal stenosis and those without subglottic stenosis. Among all samples, microbial community diversity differences were observed in scar versus nonscar regions, as well as among the various sample sites. Additionally, significant differences in community member composition was noted for tissue type (scar versus nonscar samples), etiology type (iatrogenic, idiopathic), sample site (glottis, subglottis, supraglottis, trachea), groups (iatrogenic, idiopathic, internal control, external control), sex, and individual patients. Differences in sex and individual patients are unsurprising, given the disproportionately high ratio of female to male patients represented in this population and anticipated within-subject similarity. Of more relevance are the differences found within tissue types and etiology. Within scar versus nonscar region comparisons, less diversity was observed in scar samples. This reflects similar patterns for microbial community shifts and reductions in diversity that are commonly associated with disease states in other organ systems (26, 27). Interestingly, these differences were found despite the fact that scar and nonscar region samples encompassed different etiologies and included internal controls. Thus, it is particularly meaningful that differences in scar samples were detected, despite a higher likelihood for similar microbial representation among internal controls.

Further evidence that distinct microbiota correlate to subglottic stenosis of various etiologies can be demonstrated by the differences among etiology type and groups in addition to tissue type. Given that community composition was significantly different while diversity profiles remained similar among etiologies, we infer that distinct microbial members are associated with etiology-specific pathophysiology mechanisms. This implication is particularly salient considering that these differences remain consistent even when internal controls among compared groups are included. As such, we speculate that dysbiosis of the microbiota at the injury and disease site may directly contribute to profibrotic conditions and prime the mucosa toward scar proliferation.

Within idiopathic stenosis samples, a statistically significant representation of members of the *Moraxellaceae* family, which includes the genera *Moraxella* and Acinetobacter, were found when compared with external controls. In addition to *Mycobacterium*, the presence of *Moraxella* and Acinetobacter in idiopathic scar has been detected in a prior study by Gelbard et al. and further supports a potential role of these genera in laryngotracheal stenosis pathogenesis (23). Importantly, bacteria within the genera *Moraxella* and Acinetobacter are known inflammatory pathogens within the respiratory tract. For example, Moraxellacatarrhalis has been demonstrated to contribute to exacerbations in chronic obstructive pulmonary disease (COPD) and asthma and plays a role in acute and chronic sinusitis, as well as bronchopulmonary infections in the immunosuppressed (28). Members of the genus Acinetobacter have been implicated in respiratory infections and are commonly nosocomial (29, 30). While Gelbard et al. suggested that *Mycobacterium* may be specific to idiopathic stenosis formation, differences in our results are likely a consequence of using different sample processing techniques and methodologies (31, 32).

We also assessed differences between the experimental and control groups by visualizing the samples using a principal component analysis (PCA) but did not see any discernible separation between the groups, likely due to the relatively small number of specimens in each group. However, our PERMANOVA of these groups revealed a difference in the species abundance between the idiopathic samples and external controls.

Given the broad associations among various inflammation-related respiratory conditions, it is compelling that these genera are also implicated in cases of idiopathic laryngotracheal stenosis where no apparent trigger for inflammation and scar deposition can be found. Antibiotics have been shown to be an effective therapeutic option in stenosis management, which supports the idea of a microbial component in the chronic inflammatory state and the proliferation of scar tissue associated with this pathology (33, 34). Thus, we suggest that inflammation-inducing microbial shifts can lead to pathogenesis, although it remains unknown whether the presence of pathogenic bacteria leads to host tissue inflammation or whether chronic inflammation allows for pathogen colonization and subsequent community shifts with resulting pathological scar formation.

Of particular interest is our finding of an inverse correlation between *Prevotella*, a known commensal member in the respiratory tract, and *Streptococcus*, a commonly recognized pathogen. Because commensal bacteria can benefit the host by preventing pathogen adherence (35, 36), this may explain why a decrease in the abundance of *Prevotella* coincides with an increase in members of the streptococci, which may include potential pathogens. Additionally, it is noteworthy that of the six OTUs found to be poorly represented in stenosis samples (iatrogenic, idiopathic, and internal control samples), compared with external controls, five were identified to the genus *Prevotella*. Furthermore, *Prevotella* had significantly greater abundance in external mucosal controls than in idiopathic subglottic stenosis samples, suggesting that the loss of commensal species may allow pathogens to dominate and contribute to scar formation. While it is difficult to delineate causative conclusions, the distinct lack of *Prevotella* in stenosis samples further suggests a potential interplay between commensal bacteria, *Streptococcus* proliferation, and pathogenesis. Interestingly, members of *Streptococcus* appear to play a role in pathological scar deposition in other areas of the airway (37, 38). Specifically, *Streptococcus* and several other genera have been found to be associated with idiopathic pulmonary fibrosis (IPF), a disease similarly associated with scar deposition, in comparison with nondiseased lung samples (37). Further, *Streptococcus* was determined to contribute to IPF progression (37, 38). Streptococci may, therefore, play a role in the dysregulation of extracellular matrix protein deposition that is characteristic of scar formation. Alternatively, streptococci may potentially be a factor in the overall community shift that contributes to an inflammatory state, which, in turn, could affect scarring. More research is required to define the bacterium-affiliated mechanisms of inflammation leading to fibrosis in laryngotracheal stenosis and throughout the respiratory tract, as there may be similarities in scar pathogenesis among respiratory tract fibrotic diseases.

While the idiopathic subglottic stenosis specimens demonstrated an association with *Moraxella* abundance and reduced *Prevotella* abundance, the iatrogenic subglottic stenosis specimens did not reveal any associations with microbial composition. We included this cohort as a separate etiology of subglottic stenosis with a potentially different microbial composition contributing to fibrosis. As the large majority of iatrogenic subglottic stenosis patients are critically ill and on antibiotics, we hypothesized that microbial dysbiosis could be a critical factor in predisposing patients to fibrosis at the site of the endotracheal or tracheostomy tube. The lack of a specific microbial association could be due to the heterogeneous nature of iatrogenic disease, which may be due to suprastructural cartilaginous injury and/or soft tissue fibrosis of the tracheal mucosa.

Current literature on the microbiota of the upper and lower respiratory tract supports the idea of a unified airway model due to a continuous mucosal lining and shared secretions along the airway (18, 39–41). Contrary to this paradigm, our findings show diversity and composition differences within specific sampling sites along the respiratory tract. Although a limitation of this study includes variable sample sizes among groupings, we note that of all sample sites, the glottic region appears to be less diverse and, therefore, distinct. This is particularly interesting since the glottis encompasses specialized features that differ from the rest of the airway. Not only is this region composed of stratified squamous epithelium, rather than the ciliated pseudostratified columnar epithelium that lines the trachea (15), but also the glottis functionally sustains vibratory forces for phonation that are not incurred elsewhere in the respiratory tract. These forces may also impart a separate inflammatory trigger, which could potentially influence a microbial niche population.

While this study demonstrated an association between laryngotracheal stenosis etiology and microbial composition and diversity, it is possible that the inflammation and diseased state of the trachea contributed to changes in diversity and composition. For example, frequent hospital visits and surgical procedures may place idiopathic laryngotracheal stenosis patients at higher risk for exposure to different pathogens, which may drive community shifts with an increased presence of the pathogens *Moraxella* and Acinetobacter. Another possibility is that diminished diversity is a secondary factor and does not impact the development of stenosis. More work is warranted in order to investigate the airway microbiota differences within these proximal sites as part of the overarching framework of a unified airway model, as well as to investigate if microorganism changes cause inflammation and stenosis. Distinguishing the microbial role in inflammation along the entire respiratory tract can ultimately provide valuable insights for functional differences in normal mucosal physiology and dysbiosis-affiliated disease pathogenesis.

### Conclusions.

Laryngotracheal stenosis remains a challenging and debilitating condition characterized by mucosal inflammation and scarring that requires invasive management regardless of etiology. A more thorough understanding of the triggers for mucosal inflammation that lead to scar deposition is highly beneficial for developing therapeutic modalities to improve current disease management. In this study, idiopathic stenosis samples demonstrated reduced abundance of the commensal *Prevotella* and a distinct association with members of the family *Moraxellaceae*, which includes known respiratory pathogens in the genera *Moraxella* and Acinetobacter. Community diversity differences were also identified in scar versus nonscar regions and among different sample sites. These differences may suggest dysbiosis as a direct factor for stenosis pathogenesis or that the observed microbial shift is a secondary consequence of disease. This study offers additional evidence for idiopathic stenosis having a distinct microbial composition and provides a framework for further research to delineate microbial roles in stenosis pathogenesis.

## MATERIALS AND METHODS

This study was performed in accordance with the Declaration of Helsinki and good clinical practices and was approved by the institutional review board at Johns Hopkins University to permit inclusion of sample processing at the University of Wisconsin–Madison (IRB study number NA_00078310).

### Patients and sample collection.

Participant recruitment was completed among patients being surgically treated in the operating room under general anesthesia for laryngotracheal stenosis, benign vocal fold lesions, or long-term respiratory failure requiring tracheostomy at Johns Hopkins University Hospital, Baltimore, MD. Patients without laryngotracheal stenosis (i.e., benign vocal fold lesions) were included to serve as non-tracheal disease controls. Patients under the age of 18 and women who were pregnant were excluded from this study.

A sterile tubed metal laryngoscope (universal modular glottiscope; Endocraft, LLC, Boston, MA) was introduced through the oral cavity and oropharynx to provide unobstructed access to the larynx and trachea. Up to four bacterial samples were collected per patient using a sterile minitip nylon flocked swab (ESwab 482 C; Copan Diagnostics Inc., Murietta, CA) for a total of 121 swab samples. Separate swabs were rotated along several mucosal sites per patient (supraglottis, subglottis, glottis, and trachea) to sample scar and nonscar regions. In patients with laryngotracheal stenosis, swabs were obtained from tracheal scar regions, as well as scar-free tracheal regions, to serve as a within-patient control. Swabs were immediately submerged in 1 ml of liquid Amies solution provided with the swab transport system (ESwab 482 C; Copan Diagnostics Inc, Murietta, CA) and snap-frozen on dry ice for transportation to the laboratory at the University of Wisconsin–Madison for processing. For the purposes of this study, swabs brushed from disease-free tracheal regions in patients with laryngotracheal stenosis are referred to as internal controls. Swabs collected from tracheal regions in patients without laryngotracheal stenosis are referred to as external controls.

### Sample processing.

Swabs were gently thawed at room temperature, vortexed for 10 min, and then centrifuged to condense microbial components. Approximately 970 μl of liquid Amies supernatant was removed. Pellets were resuspended into the remaining solution, and all contents were transferred into a sterile bead tube from a Mo Bio Powersoil kit (Mo Bio Laboratories, Inc., Carlsbad, CA). Total genomic DNA was extracted using a Mo Bio Powersoil kit (Mo Bio Laboratories, Inc., Carlsbad, CA) per the manufacturer’s protocol, with C2 and C3 buffers combined for extraction, and stored at −20°C until further processing. A sterile Copan ESwab was submerged in liquid Amies solution provided in the transport system and used to create a negative DNA extraction control that was processed along with all samples for sequencing. All samples were quantified using a Qubit fluorometer (Invitrogen, San Diego, CA). PCR for bacterial DNA amplification and dual-index tagging was performed using 2.5 ng of template DNA, 0.2 μl Phusion high-fidelity DNA polymerase (New England BioLabs, Ipswich, MA), and 10 μM forward and reverse universal primers flanking the V4 region of the bacterial 16S rRNA gene (42), in water to 25 μl per reaction. In addition to the negative DNA extraction control, negative PCR controls created using nuclease-free water without DNA template were incorporated with each PCR run and included in subsequent sequencing. Thermocycling conditions were as follows: initial denaturation of 98°C for 3 min, followed by 30 cycles of 98°C for 30 s, 55°C for 20 s, and 72°C for 20 s. Subsequent final extension was held at 72°C for 5 min. Gel electrophoresis for all samples and negative PCR controls was performed in 1% low-melt agarose gel with SYBR Safe (ThermoFisher, Madison, WI, USA) for confirmation of bacterial amplification and absence of contamination. Visualized samples and negative PCR controls were gel extracted on a blue light transilluminator (Clare Chemical Research, Dolores, CO) and purified using the ZR-96 ZymoClean gel DNA recovery kit (Zymo Research, Irvine, CA) to remove primer dimers and spurious PCR fragments. Samples were again quantified using the Qubit fluorometer and pooled into an equimolar library with 10% PhiX control DNA for Illumina-based sequencing on a MiSeq sequencer using a MiSeq 2 × 250 v2 kit (Illumina, San Diego, CA).

### Data analysis.

Raw sequences were processed using mothur v1.39.5 (43) by following the standard operating procedure (SOP) described by Kozich et al. (42) (http://www.mothur.org/wiki/Schloss_SOP; accessed 13 June 2017). Sequences were aligned to the GreenGenes reference database (44) for operational taxonomic unit (OTU) group assignment, and chimeras were removed using UCHIME (45). Eukaryotic and unclassifiable sequences were removed using the classify.seqs command in mothur. All remaining sequences were grouped into 97% OTUs and used for principal-component analysis.

Each data set was subsampled to 1,018 sequences to ensure even sequencing depth across samples. Samples with fewer than 1,018 sequences were omitted. OTU abundances were used to calculate Chao-1, Shannon (H’), and dominance (D) diversity indices. A nonparametric Kruskal-Wallis test with Bonferroni’s correction was used to compare the diversity indices between defined groups using SPSS Statistics 23. To test if the centroids of the defined groups were equivalent, we performed a permutational analysis of variance (PERMANOVA), using Bray-Curtis similarity, implemented in the PAST software (46). PCA was generated using STAMP (47). ANOVA with false discovery rate (FDR) correction was used to test the association of OTUs and the defined groups using STAMP. Box plots were generated using STAMP. Fisher’s exact test was used to assess for differences in prevalence of *Moraxellaceae.* Pairwise Spearman’s rank correlation (rs) among the top 15 bacterial genera was calculated in PAST. A network was built using Cytoscape 3.3.0 (48), where the edges represent correlation (rs) values of greater than 0.3 or less than −0.2 and *P* values of less than 0.05. Edge width was proportional to the modular rs values.

### Data availability.

Sequences have been deposited in NCBI under BioProject accession no. PRJNA532450. The data sets used and analyzed for this study will be made available to any qualified researcher by the corresponding authors without undue reservation on reasonable request.

10.1128/mSphereDirect.00211-19.2 FIG S2 Principal-component analysis (PCA) of all samples by group. PCA illustrating a lack of defined community clustering among etiology groups, with iatrogenic stenosis samples depicted as blue circles, idiopathic stenosis samples as red squares, internal controls as green triangles, and external control samples as yellow diamonds. Download FIG S2, EPS file, 5.4 MB. Copyright © 2019 Hillel et al. 2019 Hillel et al. This content is distributed under the terms of the Creative Commons Attribution 4.0 International license.
