# Supplementary material for: Laryngotracheal Microbiota in Adult Laryngotracheal Stenosis
Source: mSphere. 2019 May 1;4(3):e00211-19. doi: 10.1128/mSphereDirect.00211-19 (PMC6495342; doi:10.1128/mSphereDirect.00211-19)
Supplement: TABLE S1 [file mSphereDirect.00211-19-st001.pdf]

Supplemental Table S1

Swab ID	Patient ID	Group	Type	Etiology	Sample Site	Age	Gender	Smoker
S301	3	Int control	Normal	Iatrogenic	Supraglottis	59	F	Never
S311	3	Iatrogenic	Scar	Iatrogenic	Subglottis	59	F	Never
S401	4	Int control	Normal	Idiopathic	Supraglottis	45	M	Never
S501	5	Int control	Normal	Iatrogenic	Supraglottis	51	F	Former
S502	5	Int control	Normal	Iatrogenic	Supraglottis	51	F	Former
S511	5	Iatrogenic	Scar	Iatrogenic	Subglottis	51	F	Former
S512	5	Iatrogenic	Scar	Iatrogenic	Subglottis	51	F	Former
S701	7	Int control	Normal	Iatrogenic	Supraglottis	27	F	Never
S711	7	Iatrogenic	Scar	Iatrogenic	Subglottis	27	F	Never
S712	7	Iatrogenic	Scar	Iatrogenic	Subglottis	27	F	Never
S801	8	Int control	Normal	Iatrogenic	Supraglottis	44	F	Never
S802	8	Int control	Normal	Iatrogenic	Supraglottis	44	F	Never
S811	8	Iatrogenic	Scar	Iatrogenic	Subglottis	44	F	Never
S1201	12	Int control	Normal	Idiopathic	Supraglottis	45	F	Never
S1202	12	Int control	Normal	Idiopathic	Supraglottis	45	F	Never
S1211	12	Idiopathic	Scar	Idiopathic	Subglottis	45	F	Never
S1301	13	Int control	Normal	Iatrogenic	Supraglottis	62	M	Former
S1501	15	Int control	Normal	Iatrogenic	Supraglottis	22	F	Former
S1502	15	Int control	Normal	Iatrogenic	Supraglottis	22	F	Former
S1511	15	Iatrogenic	Scar	Iatrogenic	Subglottis	22	F	Former
S1512	15	Iatrogenic	Scar	Iatrogenic	Subglottis	22	F	Former
S1691	16	Ext control	Normal	None	Trachea	37	M	Never
S1692	16	Ext control	Normal	None	Supraglottis	37	M	Never
S1791	17	Ext control	Normal	None	Trachea	65	F	Active
S1792	17	Ext control	Normal	None	Supraglottis	65	F	Active
S1801	18	Int control	Normal	Iatrogenic	Supraglottis	53	M	Former
S1901	19	Int control	Normal	Idiopathic	Supraglottis	31	F	Never
S1911	19	Idiopathic	Scar	Idiopathic	Trachea	31	F	Never
S2001	20	Int control	Normal	Idiopathic	Supraglottis	36	F	Never
S2011	20	Idiopathic	Scar	Idiopathic	Trachea	36	F	Never
S2111	21	Iatrogenic	Scar	Iatrogenic	Subglottis	50	F	Active
S2312	23	Iatrogenic	Scar	Iatrogenic	Glottis	28	M	Never
S2313	23	Iatrogenic	Scar	Iatrogenic	Glottis	28	M	Never
S2601	26	Int control	Normal	Idiopathic	Supraglottis	60	F	Former
S2701	27	Int control	Normal	Iatrogenic	Supraglottis	34	F	Never
S2901	29	Int control	Normal	Iatrogenic	Supraglottis	66	F	Former
S2911	29	Iatrogenic	Scar	Iatrogenic	Glottis	66	F	Former
S3001	30	Int control	Normal	Iatrogenic	Supraglottis	17	F	Never
S3002	30	Int control	Normal	Iatrogenic	Trachea	17	F	Never

S3011	30	Iatrogenic	Scar	Iatrogenic	Subglottis	17	F	Never
S3101	31	Int control	Normal	Idiopathic	Supraglottis	35	F	Never
S3111	31	Idiopathic	Scar	Idiopathic	Subglottis	35	F	Never
S3201	32	Int control	Normal	Iatrogenic	Trachea	28	F	Former
S3202	32	Int control	Normal	Iatrogenic	Trachea	28	F	Former
S3211	32	Iatrogenic	Scar	Iatrogenic	Glottis	28	F	Former
S3212	32	Iatrogenic	Scar	Iatrogenic	Glottis	28	F	Former
S3391	33	Ext control	Normal	None	Trachea	51	F	Former
S3392	33	Ext control	Normal	None	Supraglottis	51	F	Former
S3411	34	Idiopathic	Scar	Idiopathic	Subglottis	35	F	Never
S3511	35	Idiopathic	Scar	Idiopathic	Subglottis	53	F	Never
S3601	36	Int control	Normal	Iatrogenic	Supraglottis	39	M	Never
S3611	36	Iatrogenic	Scar	Iatrogenic	Subglottis	39	M	Never
S3801	38	Int control	Normal	Iatrogenic	Trachea	33	F	Former
S3811	38	Iatrogenic	Scar	Iatrogenic	Subglottis	33	F	Former
S3901	39	Int control	Normal	Iatrogenic	Trachea	54	F	Former
S3911	39	Iatrogenic	Scar	Iatrogenic	Subglottis	54	F	Former
S4011	40	Idiopathic	Scar	Idiopathic	Subglottis	68	F	Former
S4301	43	Int control	Normal	Idiopathic	Trachea	39	F	Never
S4311	43	Idiopathic	Scar	Idiopathic	Subglottis	39	F	Never
S4511	45	Iatrogenic	Scar	Iatrogenic	Subglottis	56	F	Never
S4691	46	Ext control	Normal	None	Trachea	32	F	Never
