# Supplementary material for: Laryngotracheal Microbiota in Adult Laryngotracheal Stenosis
Source: mSphere. 2019 May 1;4(3):e00211-19. doi: 10.1128/mSphereDirect.00211-19 (PMC6495342; doi:10.1128/mSphereDirect.00211-19)
Supplement: TABLE S2 [file mSphereDirect.00211-19-st002.pdf]

Supplemental Table S2

A

PERMANOVA **Group**

Permutation N:	9999
Total sum of squares:	17.93
F:	2.083
p (same):	0.0008

**Bonferroni corrected**	Stenosis control	Idiopathic	Iatrogenic	Mucosal control
Stenosis control		0.666	0.5376	0.0114
Idiopathic	0.666		0.1332	0.006
Iatrogenic	0.5376	0.1332		0.0102
Mucosal control	0.0114	0.006	0.0102	

B

PERMANOVA **Tissue Type**

Permutation N:	9999
Total sum of squares:	17.93
Within-group sum of squares:	17.4
F:	1.778
p (same):	0.0346

**Bonferroni corrected**	Normal	Scar
Normal		0.0368
Scar	0.0368	

C

PERMANOVA **Etiology**

Permutation N:	9999
Total sum of squares:	8.79E+06
Within-group sum of squares:	8.16E+06
F:	2.255
p (same):	0.0113

**Bonferroni corrected**	Idiopathic	Iatrogenic	None
Idiopathic		0.4644	0.0054
Iatrogenic	0.4644		0.0726
None	0.0054	0.0726	

D

PERMANOVA **Sample Site**

Permutation N:	9999
Total sum of squares:	17.93
Within-group sum of squares:	16.51
F:	1.629
p (same):	0.0077

**Bonferroni corrected**	Supraglottis	Subglottis	Trachea	Glottis
Supraglottis		0.93	1	0.0426
Subglottis	0.93		0.1656	0.4392
Trachea	1	0.1656		0.3726
Glottis	0.0426	0.4392	0.3726	

E

PERMANOVA **Sex**

Permutation N:	9999
Total sum of squares:	17.93
Within-group sum of squares:	17.38
F:	1.865
p (same):	0.0246

**Bonferroni corrected**	F	M
F		0.0273
M	0.0273	

F

PERMANOVA **Smoking**

Permutation N:	9999
Total sum of squares:	17.93
Within-group sum of squares:	17.07
	1.457
p (same):	0.0545

**Bonferroni corrected**	Never	Former	Active
Never		0.2664	0.3456
Former	0.2664		0.4491
Active	0.3456	0.4491	

G

PERMANOVA **Patient**

Permutation N:	9999
Total sum of squares:	17.93
Within-group sum of squares:	6.102
F:	1.938
p (same):	0.0001

Bonferroni corrected for Individual Patient

Patient #	12	13	15	16	17	18	19	20	21	23	26	27	29	30	3	31	32	33	34	35	36	38	39	4	40	43	45	46	5	7	8
12		1	1	1	1	1	1	1	1	1	1	1	1	1	1	1	1	1	1	1	1	1	1	1	1	1	1	1	1	1	1
13	1		1	1	1	1	1	1	1	1	1	1	1	1	1	1	1	1	1	1	1	1	1	1	1	1	1	1	1	1	1
15	1	1		1	1	1	1	1	1	1	1	1	1	1	1	1	1	1	1	1	1	1	1	1	1	1	1	1	1	1	1
16	1	1	1		1	1	1	1	1	1	1	1	1	1	1	1	1	1	1	1	1	1	1	1	1	1	1	1	1	1	1
17	1	1	1	1		1	1	1	1	1	1	1	1	1	1	1	1	1	1	1	1	1	1	1	1	1	1	1	1	1	1
18	1	1	1	1	1		1	1	1	1	1	1	1	1	1	1	1	1	1	1	1	1	1	1	1	1	1	1	1	1	1
19	1	1	1	1	1	1		1	1	1	1	1	1	1	1	1	1	1	1	1	1	1	1	1	1	1	1	1	1	1	1
20	1	1	1	1	1	1	1		1	1	1	1	1	1	1	1	1	1	1	1	1	1	1	1	1	1	1	1	1	1	1
21	1	1	1	1	1	1	1	1		1	1	1	1	1	1	1	1	1	1	1	1	1	1	1	1	1	1	1	1	1	1
23	1	1	1	1	1	1	1	1	1		1	1	1	1	1	1	1	1	1	1	1	1	1	1	1	1	1	1	1	1	1
26	1	1	1	1	1	1	1	1	1	1		1	1	1	1	1	1	1	1	1	1	1	1	1	1	1	1	1	1	1	1
27	1	1	1	1	1	1	1	1	1	1	1		1	1	1	1	1	1	1	1	1	1	1	1	1	1	1	1	1	1	1
29	1	1	1	1	1	1	1	1	1	1	1	1		1	1	1	1	1	1	1	1	1	1	1	1	1	1	1	1	1	1
30	1	1	1	1	1	1	1	1	1	1	1	1	1		1	1	1	1	1	1	1	1	1	1	1	1	1	1	1	1	1
3	1	1	1	1	1	1	1	1	1	1	1	1	1	1		1	1	1	1	1	1	1	1	1	1	1	1	1	1	1	1
31	1	1	1	1	1	1	1	1	1	1	1	1	1	1	1		1	1	1	1	1	1	1	1	1	1	1	1	1	1	1
32	1	1	1	1	1	1	1	1	1	1	1	1	1	1	1	1		1	1	1	1	1	1	1	1	1	1	1	1	1	1
33	1	1	1	1	1	1	1	1	1	1	1	1	1	1	1	1	1		1	1	1	1	1	1	1	1	1	1	1	1	1
34	1	1	1	1	1	1	1	1	1	1	1	1	1	1	1	1	1	1		1	1	1	1	1	1	1	1	1	1	1	1
35	1	1	1	1	1	1	1	1	1	1	1	1	1	1	1	1	1	1	1		1	1	1	1	1	1	1	1	1	1	1
36	1	1	1	1	1	1	1	1	1	1	1	1	1	1	1	1	1	1	1	1		1	1	1	1	1	1	1	1	1	1
38	1	1	1	1	1	1	1	1	1	1	1	1	1	1	1	1	1	1	1	1	1		1	1	1	1	1	1	1	1	1
39	1	1	1	1	1	1	1	1	1	1	1	1	1	1	1	1	1	1	1	1	1	1		1	1	1	1	1	1	1	1
4	1	1	1	1	1	1	1	1	1	1	1	1	1	1	1	1	1	1	1	1	1	1	1		1	1	1	1	1	1	1
40	1	1	1	1	1	1	1	1	1	1	1	1	1	1	1	1	1	1	1	1	1	1	1	1		1	1	1	1	1	1
43	1	1	1	1	1	1	1	1	1	1	1	1	1	1	1	1	1	1	1	1	1	1	1	1	1		1	1	1	1	1
45	1	1	1	1	1	1	1	1	1	1	1	1	1	1	1	1	1	1	1	1	1	1	1	1	1	1		1	1	1	1
46	1	1	1	1	1	1	1	1	1	1	1	1	1	1	1	1	1	1	1	1	1	1	1	1	1	1	1		1	1	1
5	1	1	1	1	1	1	1	1	1	1	1	1	1	1	1	1	1	1	1	1	1	1	1	1	1	1	1	1		1	1
7	1	1	1	1	1	1	1	1	1	1	1	1	1	1	1	1	1	1	1	1	1	1	1	1	1	1	1	1	1		1
8	1	1	1	1	1	1	1	1	1	1	1	1	1	1	1	1	1	1	1	1	1	1	1	1	1	1	1	1	1	1	
